# Flavin transferase ApbE: From discovery to applications

**DOI:** 10.1016/j.jbc.2025.108453

**Published:** 2025-03-26

**Authors:** Xiaoman Fan, Marco W. Fraaije

**Affiliations:** Molecular Enzymology, University of Groningen, Groningen, The Netherlands

**Keywords:** covalent flavinylation, FMN, flavin transferase, posttranslational modification

## Abstract

ApbE is a unique, membrane-bound enzyme which covalently attaches a flavin cofactor to specific target proteins. This irreversible posttranslational modification is crucial for proper functioning of various bacterial proteins. ApbEs have also been identified in archaea and eukaryotes. This review summarizes current knowledge on the structural and mechanistic properties of this unique protein-modifying enzyme and its recent applications. The flavin transferase is typically membrane-anchored and located in the periplasm and it possesses a conserved flavin-binding domain and a catalytic domain. It recognizes a specific sequence motif of target proteins, resulting in flavinylation of a threonine or serine. For flavinylation, it depends on magnesium and utilizes flavin adenine dinucleotide as substrate to attach the flavin mononucleotide moiety to the target protein, analogous to phosphorylation. ApbE-mediated flavinylation supports critical bacterial respiratory and metabolic pathways. Recently, ApbE was also shown to be a versatile tool for selectively modifying proteins. Using the flavin-tagging approach, proteins can be decorated with flavin mononucleotide or other flavins. Furthermore, it was demonstrated that ApbE can be employed to turn natural noncovalent flavoproteins into covalent flavoproteins. In summary, ApbE is crucial for the maturation of various flavoproteins by catalyzing covalent flavinylation. While great progress has been made in understanding the role and mode of action of ApbE, there are still many bacterial proteins predicted to be flavinylated by ApbE for which their role is enigmatic. Also, exploration of the potential of ApbE as protein modification tool has just begun. Clearly, future research will generate new ApbE-related insights and applications.

## The role of covalent flavin cofactors

**Figure 1:**
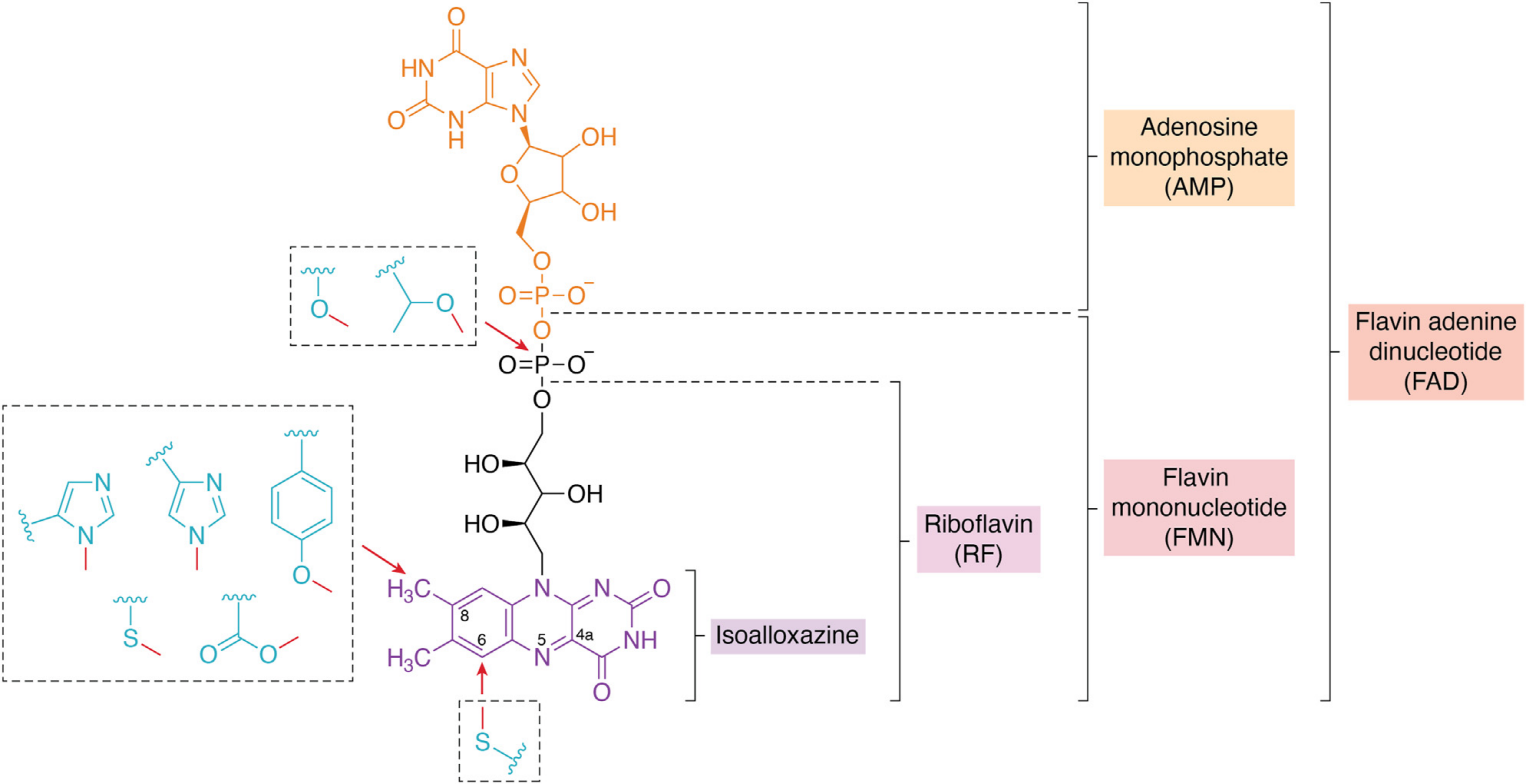
**Structures of flavins and the known flavin-protein linkages.** The redox-active isoalloxazine moiety is shown in *purple*. The RF , FMN, AMP, and FAD structures are also indicated. The type of amino acids that have been found to be form a covalent bond are shown in *cyan*, with the site of covalent attachment marked with *red arrows*. The figure was generated using ChemDraw (version 22.2.0; https://perkinelmer-chemdraw-professional.software.informer.com/). RF, riboflavin; RNF, *Rhodobacter* nitrogen fixation; FAD, flavin adenine dinucleotide.

Flavins refer to a class of natural organic compounds containing a tricyclic isoalloxazine moiety. The typical flavins are riboflavin (RF), flavin mononucleotide (FMN), and flavin adenine dinucleotide (FAD) (1). In biosynthetic routes, RF serves as the precursor of the two latter flavins: FMN is the phosphorylated form of RF, while FAD is formed by addition of an adenosine monophosphate (AMP) moiety to FMN (Fig. 1). FMN and FAD are used by a plethora of enzymes, so-called flavoenzymes, as cofactor while RF itself is rarely used as a cofactor but primarily serves as the universal precursor. These flavin cofactors play essential roles in various biological processes particularly in redox reactions where they function as cofactors for a variety of enzymes, such as glucose oxidase (2), ene reductase (3), and vanillyl alcohol oxidase (4). Because the isoalloxazine moiety is capable of undergoing one electron or two-electron reductions, allowing redox chemistry and electron transfer, and the unique reactivity of reduced flavins with molecular oxygen, flavoenzymes have evolved as crucial catalysts in various metabolic pathways and biochemical processes (5). Flavoenzymes typically utilize noncovalently but tightly bound FMN or FAD as a prosthetic group. Only a minority of flavoenzymes depend on a flavin cofactor that is dissociable during the catalytic cycle. While tightly bound in most cases through noncovalent interactions, a significant portion of flavoenzymes have been found to contain a flavin cofactor that is covalently attached to the host protein (6). The first identified covalent flavoprotein was mammalian succinate dehydrogenase, reported in 1956 by Singer *et al.* (7, 8). In the decades following this discovery, dozens of other covalent flavoenzymes have been identified (9). Nowadays, it is estimated that about 10% of all flavoproteins represent covalent flavoproteins (10).

Initially, the types of covalent flavin-protein linkages could be divided into two groups according to the linkage site, which was *via* the 8α-methylene or the C6 atom of the isoalloxazine ring, respectively (11). The majority of covalent flavoproteins contain a histidyl-linked FAD (12, 13, 14, 15). The role and mechanism of such an 8α-histidyl FAD linkage was initially mainly studied for the bacterial 6-hydroxy-D-nicotine oxidase by the Brandsch group (16). A posttranslational, self-catalytic mechanism of covalent flavinylation was discovered by reconstitution of the apo protein (17). Such mechanism was confirmed for other covalent flavoproteins containing His-FAD (18, 19), Cys-FAD (20, 21) and Tyr-FAD (22). Only recently, a new 8α-FAD linkage was discovered in a bacterial halogenase: 8α-aspartyl-FAD (23). For some covalent flavoproteins, the effect and potential role of the covalent tethering of the cofactor has been studied in detail. From work on the fungal vanillyl alcohol oxidase, it can be concluded that the covalent attachment results in an increased redox potential which assists the enzyme in oxidizing its substrates (18, 24). Later, such effect on the redox potential by covalent tethering was also found for other covalent flavoproteins and may have been the driving force for equipping some flavoenzymes with a covalent flavin. In addition to monocovalent flavin-protein linkages, some flavoenzymes have been found to contain a bicovalently bound FAD involving a 6-S-cysteinyl, 8α-N1-histidyl flavin-protein linkage. This was first recognized in 2006 when studying a fungal carbohydrate oxidase (25). In the last decade, a number of other bicovalent flavoproteins have been described from various organisms, all acting as oxidases and belonging to the same structural family of flavoproteins. While these enzymes also typically display a relatively high redox potential, the second covalent linkage does not seem to contribute significantly to increasing the redox potential (26). Many of these bicovalent flavoenzymes act on rather bulky substrates and display a moderately exposed open active site. This may be the clue of why these covalent flavoenzymes acquired a second site of attachment: it allows to have a rather open active site with a sterically fixed flavin cofactor (6).

All above-mentioned covalent flavin-protein linkages involve an attachment at the isoalloxazine moiety of the flavin cofactor. A totally different type of covalent flavinylation was reported in 2000 by Nakayama *et al.* (27). Through a thorough biochemical analysis of the membrane-bound Na^+^-translocating NADH-quinone reductase (NQR) from *Vibrio alginolyticus*, it was shown that two (NqrB and NqrC) of the six different subunits (NqrA–F) contain a covalently attached flavin cofactor, attached to a threonine (27). In a subsequent study, they established that the atypical flavin-protein linkage involved the phosphate moiety of FMN (28). Clearly, this type of covalent tethering of a flavin cofactor is fundamentally different from the above-mentioned flavin-protein linkages. The next paragraph provides an overview of the current knowledge on the occurrence, role, and mechanism of this special FMN-protein linkage.

## The discovery of the flavin transferase ApbE: connecting the dots

Flavinylation at the 8α-methylene and/or C6 of the isoalloxazine moiety in covalent flavoproteins is considered a posttranslational self-catalytic protein modification. For this covalent flavinylation process, residues around the isoalloxazine have been shown to play a crucial role. The formation of the observed FMN-threonyl linkage in NqrB and NqrC cannot proceed *via* a similar mechanism as a different part of the flavin cofactor, the phosphate group of FMN, and another type of target residue (threonine or serine) is involved. Creating such a phosphoester linkage requires another catalytic approach.

**Figure 2:**
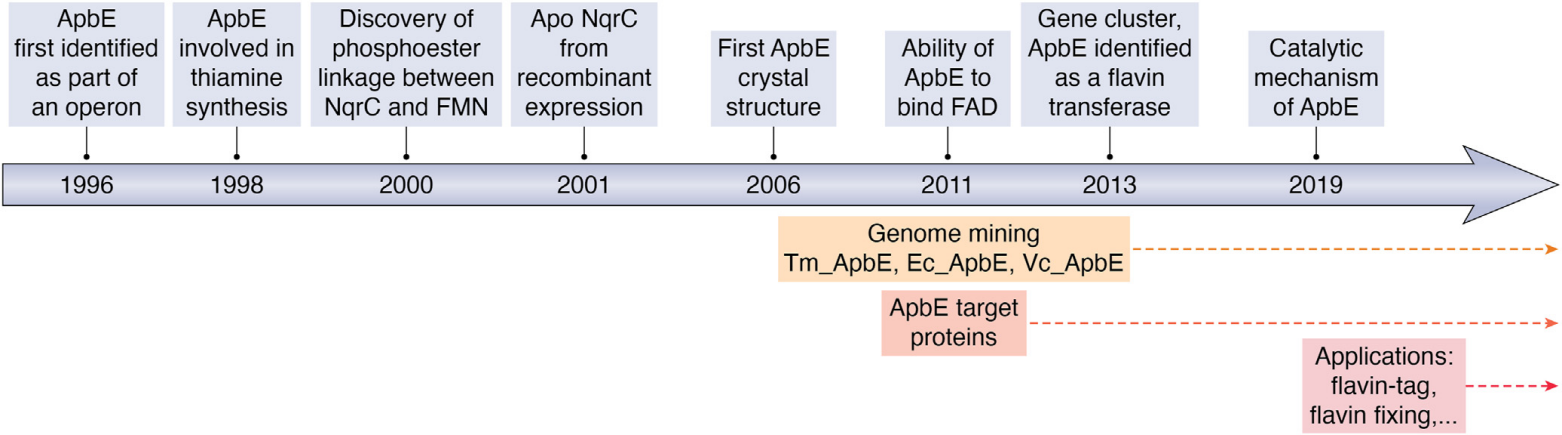
**Historic overview of progress in ApbE-related research, from discovery to applications**.

The first indication for a totally different mechanism came in 2001 from the observation that recombinant expression of NqrC from *Vibrio cholerae* in *Escherichia coli* only resulted in production of apo protein, devoid of FMN (29) (Fig. 2). A breakthrough in understanding the way FMN is covalently attached to NqrC came from the group of Bogachev in 2013 (30). Analysis of the genomic context of operons encoding for NQR in various bacteria revealed an omnipresent nearby gene: *apbE*. Also, when analyzing the operons of other bacterial protein complexes, that by then were shown to contain an FMN-protein phosphoester linkage, an *apbE* gene was invariantly found close by. Considering the fact that genes usually cluster according to their functions, they set out to probe the role of the *apbE* gene in attaching FMN to NqrC. Indeed, when coexpressing in *E. coli* the truncated versions of NqrC and ApbE, the resulting NqrC was found to contain covalent FMN. Truncating both proteins was necessary to express them as soluble cytosolic proteins. These results revealed the critical role of ApbE in attaching FMN to NqrC. The same study also demonstrated that purified apo NqrC could be flavinylated by merely incubating it with ApbE, FAD, and magnesium. This clearly established that ApbE was the missing component required for the covalent flavinylation of NqrC and similar covalent proteins. Thereafter, ApbE was identified as a flavin transferase, representing a hitherto unique posttranslational modification machinery.

**Figure 3:**
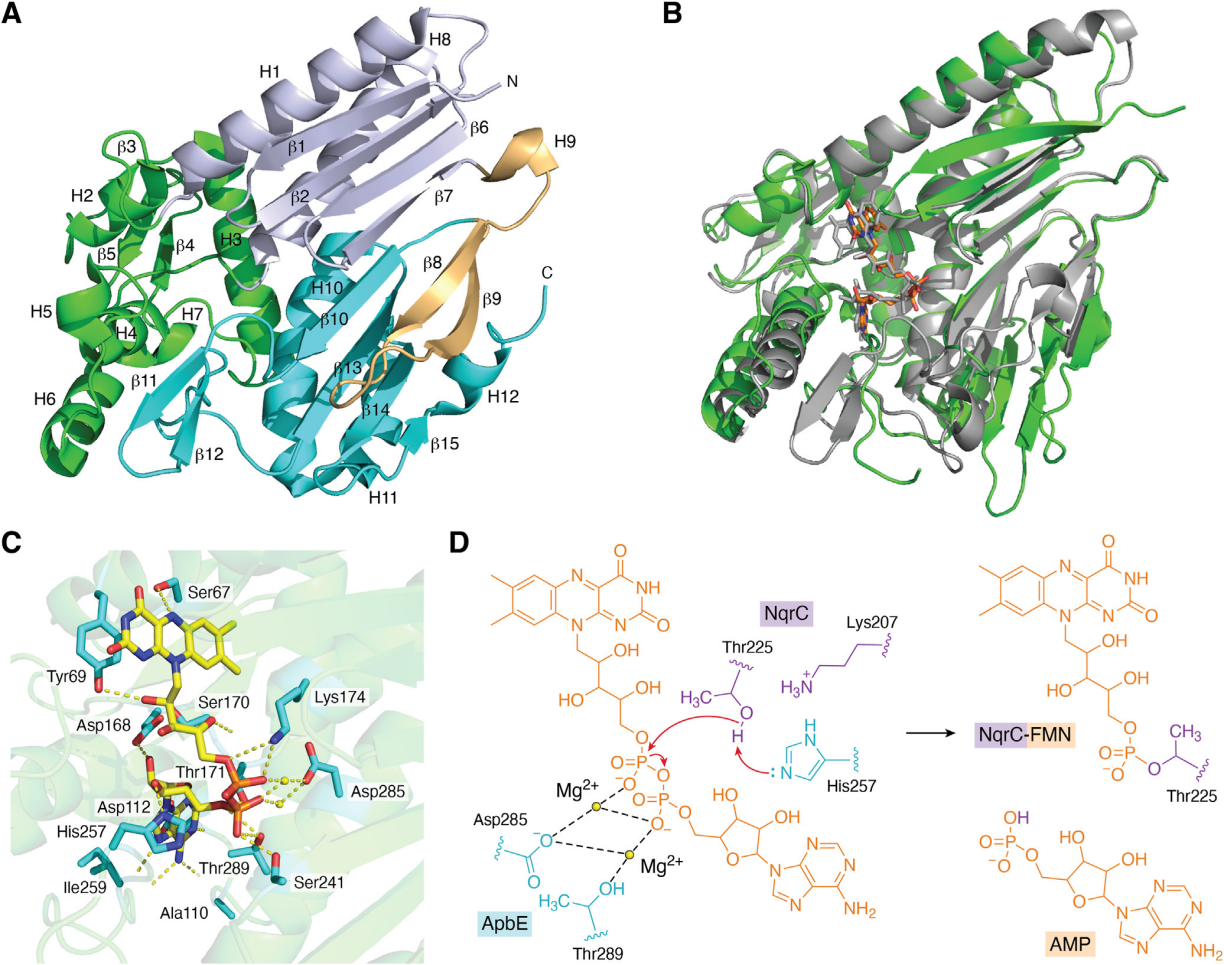
**Structural features and catalytic mechanism of ApbE.***A*, the crystal structure of Tm_ApbE (PDB ID: 1VRM) is composed of 12 α-helices and 15 β-strands (labeled). It was colored by *blue white* (N-terminal T-fold domain), *cyan* (C-terminal T-fold domain), *light orange* (linker region), and *green* (helical domain). *B*, an overlay of structure of the flavin transferase from *V. cholerae* ((PDB ID: 6NXI, in *green* and FAD in *orange* C-atoms) and the AlphaFold3 (https://alphafoldserver.com/) predicted structure of the flavin transferase domain (residue 53–343, in *gray*) of the fumarate reductase from *L. pyrrhocoris* (45). Except for a similar overall structure, FAD binding is also predicted to be similar. *C*, the key residues around FAD in the active site of the flavin transferase from *V. cholerae* (PDB ID: 6NXI). Figures generated using PyMOL (109) (https://www.pymol.org/). *D*, the proposed catalytic mechanism of Vc_ApbE (54). Figure generated using ChemDraw (version 22.2.0). FAD, flavin adenine dinucleotide.

With the realization that ApbE acts as a flavin transferase, finally the role of this bacterial protein was elucidated. The ApbE-encoding gene was first identified in 1996 as part of an operon that was linked to an alternative pyrimidine biosynthetic pathway in *Salmonella typhimurium* (31). Based on its clustering with genes involved in metabolic routes, it was suggested to catalyze a step in the synthesis of thiamine (32) and thought to play a role in iron-sulfur cluster biosynthesis (31, 32, 33). The studies established that ApbE was a lipoprotein anchored in inner membrane of periplasmic. An N-terminal signal peptide sequence results in transport to the periplasm while attachment of a lipid assures membrane anchoring. As a membrane-bound protein, it installs covalently bound FMN on secreted target proteins. In 2006, the first crystal structure of an ApbE from the bacterium *Thermotoga maritima* was reported (Fig. 3*A*) (34). At that time, the function of ApbE was not yet established. Even though an unidentified ligand was observed in the crystal structure, no conclusions could be drawn considering the function of the protein. It illustrates that ApbE has a unique structure, not showing similarity with enzymes displaying a similar activity, for example, kinases. Only in 2011, the first experimental evidence was reported on the ability of ApbE from *Salmonella enterica* to bind FAD (35). When expressed as His-tagged protein, lacking the N-terminal lipoprotein signal peptide, a slightly yellow-colored protein was obtained. The chromophore was identified as FAD and it was found that about 10% of the protein contained the flavin cofactor. The protein could be fully reconstituted with FAD and crystallized. The elucidated crystal structure finally established that ApbE has a defined binding pocket for FAD. The structure revealed that it belongs to the tunnel-fold (T-fold) superfamily. Members of this family are often involved in the biosynthesis of purines and pterins. Clearly, ApbE has another function and its structural features are distinct from other flavoprotein families. In the same study, it was also verified by generating a mutant that lost the ability to bind FAD, that the FAD binding is essential for its *in vivo* function. While these structural insights hinted to a role of FAD in the functioning of AbpE, its exact physiological role remained enigmatic. This changed in 2013 when the seminal paper of the Bogachev group was published that revealed that AbpE acts as a flavin transferase (Fig. 2).

In the last decade, more details on the role and function of ApbE and homologs thereof have been revealed. In addition to a role as flavin transferase, the Norgard group has found that some ApbEs display significant FAD hydrolase activity (36). It has been suggested that such activity, generating FMN from FAD in the periplasm of bacteria, serves a role in flavin homeostasis. Mutagenesis revealed that the switch between flavin transfer or hydrolysis may reside in just one point mutation (37). The importance of such hydrolytic activity of ApbEs and also the pathways by which FAD reaches ApbE are still obscure. Furthermore, it has become clear that many extracellular bacterial proteins are decorated with covalent FMN by ApbEs. Analysis of bacterial genomes indicates that approximately 50% of the sequenced bacterial genomes contain genes encoding proteins targeted by ApbE for flavinylation (38). It is predicted that some of these covalent flavoproteins will contain >10 FMNs covalently attached. For most of these multiflavinylated proteins, their physiological functions remain enigmatic. In addition to being present in many bacteria, ApbE-encoding genes also have been identified in the genomes of some archaea (such as *Desulfovibrio vulgaris*) and pathogenic eukaryotes (such as *Leptomonas pyrrhocoris*, *Leishmania infantum,* and *Trypanosoma brucei*) (39, 40, 41). A glycosomal fumarate reductase in *T. brucei* was recently found to be covalently flavinylated by a ApbE fused to the reductase (41).

While ApbE was discovered by having a role in the biosynthesis of thiamine, it has become clear that it is not directly involved in synthesis of a product intermediate of the respective pathway. It appears that thiamine auxotrophy in ApbE knock-out (KO) strains is due to a negative effect on the activity of 4-amino-2-methyl-5-hydroxymethylpyrimidine monophosphate synthase (ThiC), which catalyzes a crucial step in the biosynthesis of thiamine pyrophosphate. This may be due to the fact that ThiC contains an essential iron-sulfur cluster and there is strong evidence that ApbE has a role in iron-sulfur clusters biosynthesis (32, 42, 43). The exact role of covalent flavinylation in this process remains to be elucidated.

Clearly, ApbE-type flavin transferases have evolved to take up various roles in nature. In this review, we focused on providing an overview concerning the current knowledge on the molecular properties and applications of ApbE.

## Structure and mechanism of ApbE

### Structural features

Most ApbEs characterized thus far are lipoproteins harboring a lipid anchor at their N terminus. The full-length ApbE contains an N-terminal lipoprotein secretion signal which targets a protein to the secretion machinery that will first attach a lipid to a conserved cysteine (Cys17 in ApbE in *V. cholerae*) after which the protein is secreted to the periplasm. The lipid assures tight binding to the membrane. Already in one of the first studies on ApbE, it was shown that the specific localization in the periplasm is essential for the function of the flavin transferase. A cytosolically expressed ApbE, lacking the first 20 N-terminal residues, could not support growth of an ApbE KO strain in absence of thiamine (33). Interestingly, it was shown that the lipid anchor was not essential to restore growth of the mutant strain in absence of thiamine. Expression of ApbE in the periplasm, using a signal peptide that avoids formation of a lipid anchor, allowed growth in absence of thiamine. This is a bit puzzling since gram-negative bacteria have an outer membrane that would prevent ApbE from being secreted. The rationale behind the need for membrane association may lay in the process of FAD delivery to ApbE (*vide infra*). Most biochemical studies have used truncated versions of ApbE which allows cytosolic (over)expression of the flavin transferase. These truncated versions are typically fully functional, again confirming that the lipid anchor is merely there for attaching it to the membrane. While most ApbEs from bacteria and archaea seem to be secreted, also cytosolic ApbEs exist. In the predicted proteome of *Klebsiella pneumoniae,* two AbpEs were identified (44). While one contains the typical N-terminal lipoprotein secretion signal, the other ApbE lacks a signal peptide. It was shown that the cytoplasmic flavin transferase was essential for covalent flavinylation of a soluble fumarate reductase in this bacterium. Other examples of intracellular flavin transferases are the recently discovered ApbEs from *L. pyrrhocoris* and *T. brucei* (45). Intriguingly, in these studied eukaryotes, the AbpE forms an N-terminal domain of fumarate reductase (41, 45). The ApbE domain is fused to its target flavoenzyme which needs to be flavinylated. While no experimental structure is available for a eukaryotic flavin transferase, the sequence identities of these ApbE domains show very low sequence identity (<25%) with bacterial ApbEs. The structure for the N-terminal flavin transferase domain of the fumarate reductase from *L. pyrrhocoris* predicted by AlphaFold (46) reveals that it is very similar when compared with the bacterial experimental ApbE structures (Table 1 and Fig. 3*B*). This suggests a conserved mechanism and function across bacteria, archaea, and eukarya.

**Table 1:** ApbEs for which crystal structures have been elucidated

Organism	Oligomeric state	PDB ID	Resolution (Å)	Length (residues)	Year of publication (reference)
*Thermotoga maritima*	Monomer	1VRM	1.58	352	2006 (34)
*Salmonella enterica*	Tetramer	3PND	2.75	337	2011 (35)
*Treponema pallidum TP0796*	Dimer	4IFU	1.83	340	2013 (36)
*Escherichia coli K-12*	Dimer	4XGV	1.83	351	2016 (37)
*Pseudomonas stutzeri*	Tetramer	5MGY	2.60	332	2017 (47)
*Vibrio cholerae*	Dimer	6NXI	1.61	328	2019 (54)
*Listeria monocytogenes*	*Not determined*	7F39	1.89	340	2022 (113)

Most structural insights come from studies on bacterial ApbEs. This has revealed that the oligomeric states of bacterial ApbEs vary considerably. While other T-fold proteins form large oligomers to form a tunnel (hence the name T-fold), ApbEs exist in relatively small oligomers: monomers, dimers, and tetramers (Table 1) (47). Here, it is worth noting that the oligomeric states have been determined based on the N terminally truncated soluble proteins. It cannot be excluded that, when anchored to the membrane, the ApbE forms higher oligomers.

In 2006, the first crystal structure of an ApbE was reported: ApbE from *T*. *maritima* (Tm_ApbE) (34). High-quality crystallographic data (1.58 Å resolution) revealed density of a bound ligand. With the current knowledge, the ligand observed in the crystal structure can be easily identified as ADP, occupying the binding pocket of the AMP moiety of FAD. Clearly, the enzyme has a high affinity for ADP, being copurified and cocrystallized. Full-length Tm_ApbE encompasses 352 amino acids but for expression of soluble intracellular protein, a truncated version in which the first 39 N-terminal residues were removed, was expressed for crystallization studies. The elucidated structure revealed that the Tm-ApbE structure is composed of 12 α-helices and 15 β-strands (Fig. 3*A*), forming three structural domains. The N-terminal (residues 40–87 and 191–226) and C-terminal (residues 254–352) domains show high structural similarity, both representing a T-fold topology. The T-fold is composed of an antiparallel β sheet (four strands) and two α antiparallel helices positioned between the second and third strand (ββααββ) (48, 49). A short stretch of residues links the two T-fold domains. Apart from those, there is a mainly helical domain (residues 88–190) inserted between helix1 and helix8 in the N-terminal domain (34).

In recent years, a large number of other ApbE crystal structures have been reported (Table 1). These have shed light on the molecular functioning of these bacterial flavin transferases. The crystal structure of ApbE from *Pseudomonas stutzeri* (Ps_ApbE) and ApbE from *V. cholerae* (Vc_ApbE) have a high similarity compared with that of Tm_ApbE. They all share a quite similar overall structure. The RMSDs when these three ApbE structures are compared are only 1.3 to 2.1 Å. Differences are mainly found in the linkage region between the two T-fold domains and the C-terminal domain, while the core T-fold structures remain conserved, reflecting their essential role in ApbE’s function. The structural characteristics of ApbE include a mostly hydrophobic central cavity for ligand coordination, accommodating FAD, AMP, ADP, ATP, and Mg^2+^ ion. FAD binds to the central cavity of ApbE in a bent conformation, in which the adenine moiety is deeply embedded in the hydrophobic pocket and the isoalloxazine ring remains exposed outside the pocket. As a Mg^2+^-dependent enzyme, 2 Mg^2+^ ions are coordinated with the phosphate groups, potentially stabilizing the binding of FAD and facilitating its hydrolysis (Fig. 3*C*) (50, 51). Similar to FAD, AMP (the hydrolysis product of ApbE), ADP, and ATP, all bind to ApbE in a curved conformation (52).

### Catalytic mechanism

In the FAD binding pocket of ApbE, hydrogen bonds are formed between FAD and surrounding residues (Fig. 3*D*). However, most of these key residues in the catalytic center are not conserved, as indicated by their low sequence identity, except for Ser 241, His257, and Asp285 (52). This lack of conservation may explain the differences in substrate specificity. Using the ApbE from *V. cholerae* as example, the hydrogen bond between His257 and the 3′-OH of FAD locks the FAD inside the pocket. Replacing His257 with glycine does not lead to a significant change in the structure, but it can affect FAD binding and decreases the flavin transferase activity (53). The *K*_*m*_ of WT and mutant H257G toward FAD is 0.1 and 0.2 μM, respectively. It has been proposed that the catalytic mechanism of Vc_ApbE follows a random sequential mechanism (54). In the first step of the reaction, FAD and NqrC bind in a random order and form a ternary complex. The coordination of Mg^2+^ ions is supposed to mediate the binding of FAD through interactions with the oxygen atoms of the phosphate groups (Fig. 3*D*). When both FAD and NqrC are bound, Lys207 of NqrC lowers the pKa of Thr225. His257 subsequently acts as a general base, removing a proton from Thr225. The deprotonated Thr225 then performs a nucleophilic attack on the pyrophosphate group of FAD, forming a phosphoester bond with FMN, with AMP being the other product after the flavin-transfer process is finalized (55). The mechanism is consistent with other transferases, including serine proteases (56) and adenylytransferase (57, 58). In 2022, a slightly alternative catalytic mechanism of FMN transferase (FmnB) from *Listeria monocytogenes* was proposed by Zheng *et al.* (52). In this case, they identified a similar catalytic triad center (Asp301-Ser257-His273) in FmnB, where the corresponding residues (Asp285-Ser241-His257) can also be found in Vc_ApbE. The identification of His257, acting as a general base, is shared by both mechanisms. However, FmnB has been shown to contain another key residue, Arg262, which interacts with His273 in FmnB and also is involved in binding FAD. Such additional active site residue is not present in Vc_ApbE (52).

### Flavinylation motif

**Figure 4:**
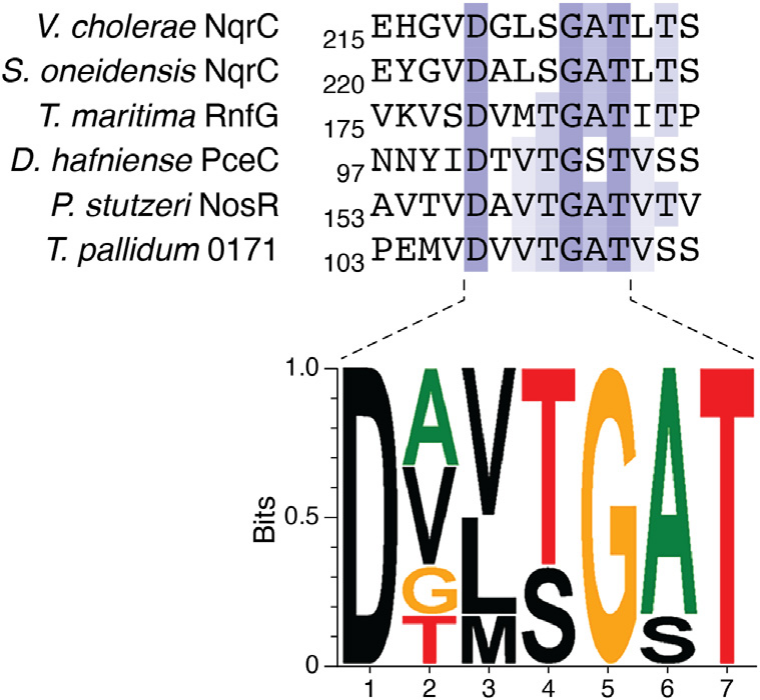
**Partial sequence alignment of flavinylated proteins containing the ApbE-recognized motif.** Vc_NqrC and So_NqrC represent the NqrC from *V. cholerae* and *Shewanella oneidensis*, respectively. Tm_RnfG is the G subunit of Rnf complex from *T. maritima*. Dh_PceC is the C subunit of the tetrachloroethene (PceC) reductive dehalogenase from *Desulfitobacterium hafniense*. Ps_NosR is the NosR from *P. stutzeri*. Tp_0171 is the *T. pallidum* periplasmic lipoprotein. The figure was generated using Jalview (110) (https://www.jalview.org/) and WebLogo (https://weblogo.threeplusone.com/) (111, 112). RNF, *Rhodobacter* nitrogen fixation.

ApbEs recognize a certain protein sequence motif and transfer the FMN moiety of FAD to the C-terminal threonine or serine of the motif. The minimal length of this flavinylation motif is only seven residues, as verified by Tong *et al.* (59). This sequence motif starts with a strictly conserved aspartate and ends with the serine or threonine to which the FMN is attached: D-x-x-[S/T]-G-A-[S/T]) as illustrated in Figure 4. In a study conducted by Bertsova *et al.*, the effects of single substitutions in the flavinylation sequence motif were examined using the FMN-containing fumarate reductase from *K. pneumoniae* as model flavoenzyme (60). They confirmed that substituting the FMN-linking threonine with an alanine completely abolished flavinylation, while the replacement of the conserved aspartate with alanine had only minor effects. The substitution of conserved glycine to alanine and alanine to valine caused a lower degree of flavinylation (∼50%). Additionally, in other studies, it was shown that ApbE can also recognize and attach FMN to a serine (41, 45). Yet, replacement of threonine to serine resulted in a 10 to 55% decrease in flavinylation degree. It indicates that ApbE has a preference for threonine over serine (59).

### ApbE inhibitors

AMP, the product of the ApbE-catalyzed flavinylation process, is an ApbE inhibitor (36, 51, 53). Fang *et al.* further identified that it acts as a mixed-type inhibitor with respect to NqrC, with inhibition constants in the micromolar range (54). The data suggest that AMP can bind to both the free enzyme and the enzyme–substrate complex. Interestingly, at high concentrations (>10 mM), AMP seems to activate the enzyme by binding to a regulatory site, indicating a complex regulatory role of AMP depending on its concentration. ADP, an analog of AMP, has been shown to enhance the activity of Vc_ApbE. Conversely, ADP exhibits strong inhibitory effects on FmnB from *L*. *monocytogenes* (52). Clearly, AMP and its analogs have multifaceted roles in modulating the activities of ApbEs. These effects are mediated through complex interactions involving the phosphate groups, the enzyme active sites, and possibly the Mg^2+^ ions, highlighting the potential for developing targeted inhibitors or activators based on these interactions.

Owing to its wide distribution among many pathogenic species, ApbE is a promising target for the development of next-generation antibiotics. And potent ApbE inhibitors could serve as effective antimicrobials by disrupting the function of key proteins it normally equips with the essential FMN cofactor. For example, Na^+^-NQR, a primary Na^+^ pump in aerobic pathogens, is a typical target of ApbE and has already been explored as a potential antibiotic target (61, 62). Inhibiting ApbE could prevent the proper assembly of Na^+^-NQR, leading to a nonfunctional enzyme complex, disrupting sodium ion transport, and ultimately exerting an antibacterial effect.

### ApbE activity assays

Studies aimed at investigating or engineering ApbEs require reliable methods to measure activity. As the primary function of ApbE is to covalently attach FMN to a target protein, measuring its activity typically involves assessing the levels of FMN incorporation or FAD hydrolysis. Here, we summarize the published technical approaches to monitor ApbE activity.

The method based in monitoring FAD hydrolysis was validated by Forti and Sturani (63) and later adapted by Deka (36). The approach leverages the fact that the fluorescence intensity of FMN is higher than that of FAD free in solution. This is due to the fluorescence quenching effect of the adenine moiety interacting with the isoalloxazine moiety of FAD. During the catalysis, ApbE hydrolyzes FAD into FMN and AMP. By monitoring the increase in fluorescence, the activity of ApbE can be followed. In this approach, typically 1.0 μM ApbE is incubated with 5 mM MgCl_2_ at 37 °C for 10 min and subsequently incubated for another 5 min after adding 10 μM FAD. For flavin fluorescence detection, excitation and emission wavelengths are set at 430 nm and 510 nm, respectively. A control reaction mixture without enzyme serves as the background. This fluorescence-based method provides a straightforward means to evaluate ApbE activity by monitoring the increase in FMN fluorescence. Despite its convenience, the method has limitations concerning accuracy and it does not account for ApbE-bound FAD.

The activity of ApbE can also be monitored by measuring the flavinylation of a target protein. For this, NqrC is often used as it can be easily produced as apo protein. The flavinylation can be detected in-gel upon SDS-PAGE of the protein due to the fluorescence of FMN when exposed to UV light (27, 53). The reaction is usually performed using 3 mg mL^−1^ NqrC, 1 mg mL^−1^ ApbE, 10 μM FAD, 1 mM EDTA, 10 mM MgCl_2_, and incubated at 30 °C for 3 h. Samples are taken at different time points by adding SDS-PAGE loading buffer. Fluorescence intensity in gels can be analyzed using imaging software (64). This approach provides a relatively simple method to follow ApbE activity.

In 2019, Fang *et al.* introduced a spectrophotometric method to measure flavin transferase activity of ApbE by exploiting the absorbance differences between free FAD and covalently bound FMN (54). This method takes advantage of the distinct absorbance spectra of these two flavin cofactors, particularly noting an isosbestic point at 366 nm and different absorbance at 395 nm. This spectrophotometric method offers a precise and cost-effective approach to measure the flavin transferase activity of ApbE. By monitoring the absorbance at 395 nm, accurate and real-time kinetic data can be obtained. This method is advantageous over the above-mentioned fluorescence and gel electrophoresis methods in terms of accuracy, simplicity, and cost, making it a preferred choice for studying ApbE activity.

## ApbE target proteins

The flavin transfer activity of ApbE is crucial for functionalizing a variety of essential bacterial respiratory enzymes. ApbE-flavinylated FMN-binding domains have been found in many confirmed or predicted extracytosolic electron transfer systems, including Na^+^-NQR, *Rhodobacter* nitrogen fixation (RNF), nitrous oxide reduction, organohalide reduction, and extracellular electron transfer. These systems play significant roles in bacterial respiration and energy conversion processes. Here, we provide a condensed overview of several well-studied enzyme systems that have been shown to be processed by ApbE.

### Na^+^-translocating NADH-quinone reductase

**Figure 5:**
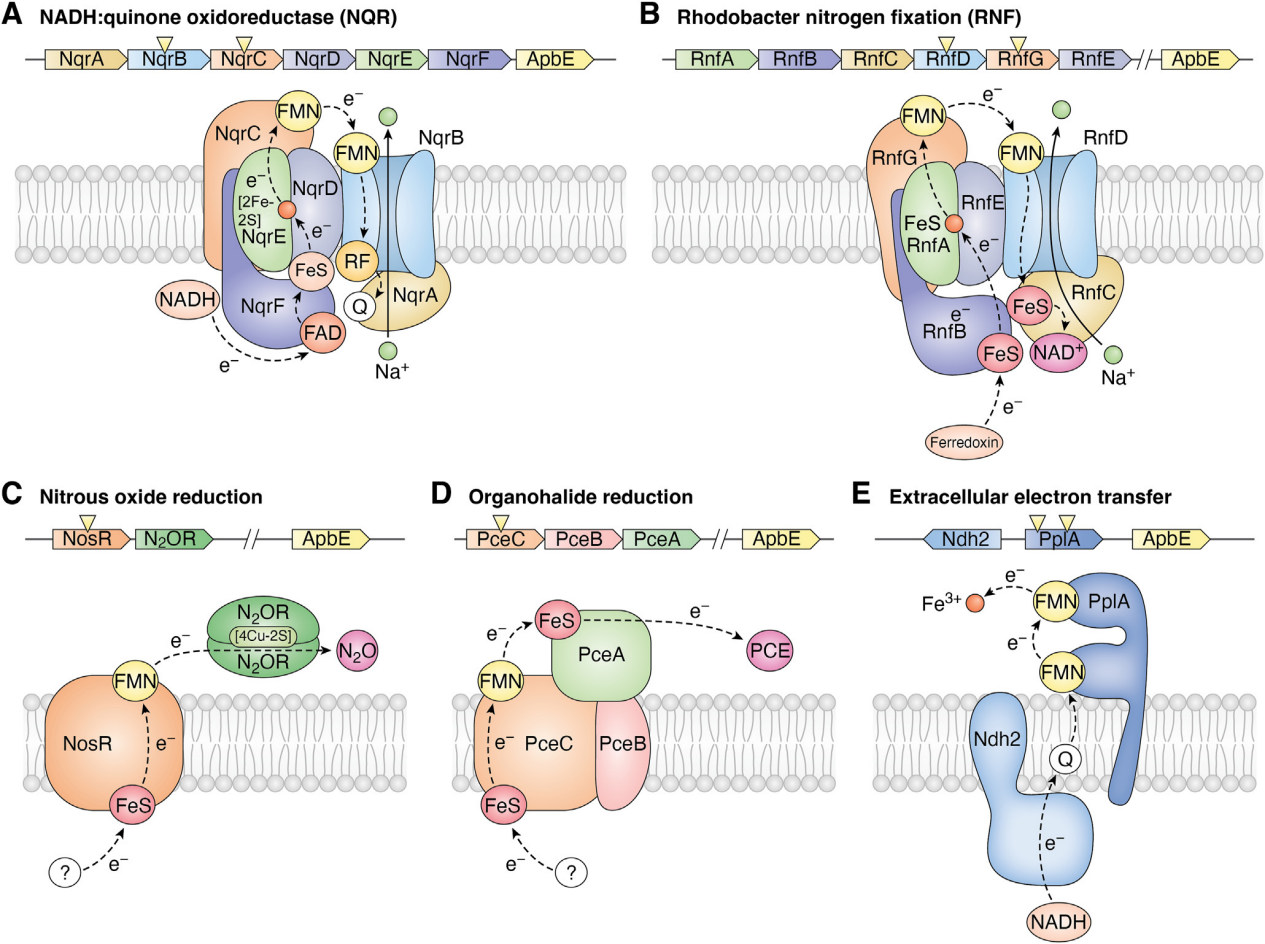
**ApbE-flavinylated proteins in characterized extracytosolic electron transfer systems.** The ApbE-flavinylated subunits in the gene clusters are labeled with *yellow triangles*. The respective FMN-bound domains or subunits are in *yellow*. The *black arrows* trace the proposed electron transfer pathways. Figure adapted from (38). *A*, NADH: quinone oxidoreductase (NQR), (*B*) *Rhodobacter* nitrogen fixation (RNF), (*C*) nitrous oxide reduction, (*D*) organohalide reduction, and (*E*) extracellular electron transfer. FMN, flavin mononucleotide.

Na^+^ is the second major coupling ion at membranes after protons, and sodium-motive force contributes to virulence and plays an important role in many pathogenic bacteria. A prominent example is the pathogenic *V. cholerae*, which relies on a respiratory NADH dehydrogenase (Na^+^-NQR) in the inner membrane to achieve the sodium ion cycle (65). The six subunits of Na^+^-NQR contain a unique set of cofactors. As shown in Figure 5*A*, the peripheral NqrF contains a noncovalently bound FAD, a 2Fe-2S cluster, and NADH. NqrDE has a 2Fe-2S cluster, NqrC has a covalently bound FMN, and NqrB has another covalently bound FMN, a noncovalently bound RF, and a ubiquinone-8 in NqrA (66, 67, 68, 69). In fact, NqrB is the only enzyme known that uses RF as cofactor. Nqr catalysis involves transfer of electrons upon oxidation of NADH, which are proposed to transfer from FAD to 2Fe-2S in NqrF, then to 2Fe-2S in NqrDE, to periplasmic FMN of NqrC, to FMN in NqrB, to RF in NqrB, and finally to ubiquinone-8 in NqrA (70, 71). Since the formation of the two covalent FMNs in NqrB and NqrC is facilitated by ApbE, the ApbE gene downstream of the Nqr operon is strictly required for the full maturation of NQR and indirectly affects many regulatory networks of *V. cholerae*.

### *Rhodobacter* nitrogen fixation

Rnf was originally discovered as an essential enzyme in nitrogen fixation in *Rhodobacter capsulatus*, and hence named as RNF protein (72, 73, 74). Nowadays, it is clear that Rnf genes are widespread in bacteria, including *V*. *cholerae, E*. *coli, Azotobacter vinelandii, Porphyromonas gingivalis, Clostridium tetanomorphum, Acetobacterium woodii,* and *Methanosarcina acetivorans*. It also shows significant sequence homology toward Na^+^-NQR. The superfamily of Rnf and Na^+^-NQR exhibits a high degree of functional variability, because Na^+^-NQR is pumping the Na^+^ outward, while the Rnf complex pumping Na^+^ inward or outward (75). Rnf is an assembly of seven units (RnfA, RnfB, RnfC, RnfD, RnfG, RnfE, and RnfH) and harbors several flavin cofactors and Fe-S centers (Fig. 5*B*). RnfD and RnfG harbor a covalently bound FMN. The Rnf complex from *T*. *maritima* uses reduced ferredoxin as the electron donor, and electrons follow a U-shaped electron transfer path to NAD^+^ (Fig. 5*B*) (76, 77). Additionally, the reaction is reversible, and an ion gradient can drive the endergonic reduction of ferredoxin using NADH as the reductant (78). The RnfD and RnfG each with ApbE-flavinylated FMN are responsible for transferring electrons. Bertsova *et al.* also confirmed that when these subunits are not flavinylated, nitrogen fixation is impaired (79).

### Nitrous oxide reductase

As a potent greenhouse gas, nitrous oxide (N_2_O) is kinetically inert to decomposition (80). Although several enzymes reported can catalyze its reduction, nitrous oxide reductase (N_2_OR) is the most efficient one for this purpose (81, 82, 83). It is a soluble dimeric enzyme located in periplasm and exists in many genera of bacteria, catalyzing the conversion of N_2_O to dinitrogen (N_2_) during microbial denitrification (Fig. 5*C*). The enzyme requires two copper sites in each monomer, CuA and CuZ, for activity. In the *P. stutzeri*, N_2_OR is encoded by the nosZ gene within the nos gene cluster (nosRZDFYL) (47). The nos gene cluster is involved in maintaining the fully functional N_2_OR. NosR is a transmembrane protein with an N-terminal flavin-binding domain located in the periplasm and is thought to act as the electron donor for the reducing activity of N_2_OR (84). Deletion of the flavin-binding domain in NosR results in a dramatic decrease or complete loss of N_2_O-reducing activity. Zhang *et al.* demonstrated that the ApbE from *P*. *stutzeri* is essential for N_2_OR maturation *via* covalent flavinylation of a threonine residue in NosR (47).

### Reductive dehalogenases

Organohalide respiration is a respiratory metabolism that conserves energy and is utilized in many anaerobic bacteria (85). The reductive dehalogenase (Rdh) is a cobalamin-dependent enzyme which is indispensable for the catalytic dehalogenation by organohalide-respiring bacteria (86, 87). Various electron transport models of Rdh have been reported, reflecting different strategies according to the phylogeny and sources of the bacteria (88). In 2018, Buttet *et al.* discovered that the C subunit of the organohalide respiration gene clusters (RdhC) may be the missing redox protein between the quinol pool and the Rdh (50). Specifically, the C subunit of the tetrachloroethene reductive dehalogenase (PceC), also called RdhC, binds FMN covalently at Thr168. The proposed electron transfer is shown in Figure 5*D*. In line with this, it was demonstrated that the reconstitution of PceC *in vitro* could be performed using ApbE. It was also shown that attachment of FMN had a significant positive effect on the folding and solubility of the recombinant FMN-binding domain (50).

### Extracellular electron transfer

Extracellular electron transfer (EET) describes the bioelectrochemical processes in which electrons are transferred from the cytosol to the exterior of the cell (89, 90). As a gram-positive bacterium, *L*. *monocytogenes* generates a significant electric current with a growth substrate, revealing a novel electron transport chain. The genetic basis of EET activity has been studied and several enzymes involved in this process have been identified (91). The neighboring gene in the EET locus, PplA, facilitates electron transfer to extracellular electron acceptors *via* its covalently bound FMN (Fig. 5*E*) (92). Here, the flavin transferase FmnB catalyzes the posttranslational flavinylation of a threonine of PlpA, which is essential for its maturation (91). Thus, FmnB indirectly promotes the EET process in *L. monocytogenes.*

Intriguingly, Méheust *et al.* predicted, through a thorough sequence analysis study, that multiple extracytosolic proteins may contain a large number of covalently bound FMN molecules. In recent study, we characterized two bacterial multi-FMN-containing proteins: a three-FMN-binding protein from *Streptomyces azureus* and a four-FMN-binding protein from *Clostridiaceae bacterium*, as predicted through sequence analysis (93). Soluble, monomeric, and multiflavinylated proteins were successfully obtained when coexpressed with ApbE as N terminally truncated proteins. Their N termini were predicted to contain a secretion signal and a transmembrane segment. Soluble three-FMN-binding protein from *Streptomyces azureus* exhibited a relatively high redox potential of −184 mV and was able to use dioxygen as electron acceptor. Such oxidase activity may have relevance for its physiological role. Both experimental data and predictions suggest that multi-FMN binding proteins may play a role in extracellular long-distance electron transfer pathways, similar to those involving multi-heme cytochromes (38). Yet, the exact roles of such multiflavinylated proteins remain enigmatic.

## Applications

### Flavin-tagging

**Figure 6:**
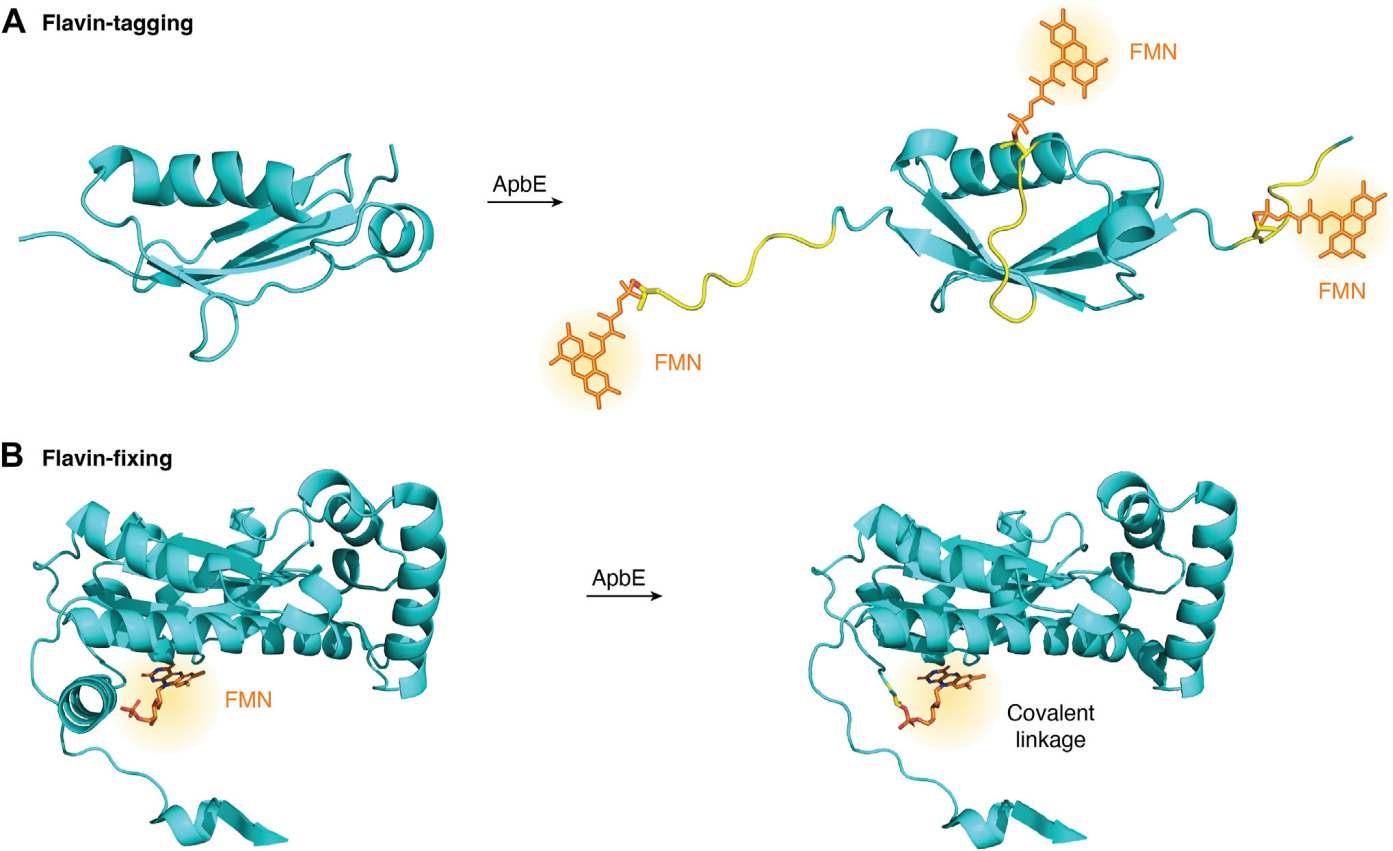
**Applications of ApbE in bioconjugation and protein engineering.***A*, flavin-tagging. SUMO is shown as an example target protein. The introduced motif recognized by ApbE is shown in *yellow*, and the structure was predicted by AlphaFold (46) (https://alphafoldserver.com/). *B*, flavin-fixing. The nitroreductase from *Bacillus tequilensis* was used as target protein (*cyan*), and the FMN is shown in *orange*. The figure was generated by PyMOL (109) (https://www.pymol.org/).

Site-specific protein labeling is a valuable method for directly visualizing and/or functionalizing specific proteins, both *in vitro* and *in vivo* (94). With the discovery of GFP (95), the field of protein labeling made significant progress, and numerous molecular tools have been developed for selective protein labeling each with its pro's and con's. Enzyme-based protein labeling is usually based on a specific peptide sequence to form a “tag” that can be recognized and/or modified. Typical enzyme-based bioconjugation examples include biotin ligase (AP-tag) (96), transglutaminases (97), lipoic acid ligase (LAP-tag) (98), and tubulin tyrosine ligase (Tub-tag) (99). But most of these approaches suffer from one or more drawbacks such as limited biocompatibility and reliance on complex chemical probes. In nature, ApbE specifically recognizes the flavinylation sequence motif and attaches an FMN to a targeted threonine merely using FAD as substrate. The UV/visible absorbance and fluorescence features of this posttranslational modification were first exploited for the subcellular localization the ApbE-flavinylated NqrC in mammalian cells (100). In 2021, we developed a generic and convenient method of site-specific labeling of proteins using ApbE (59). This method is referred to as flavin-tagging, as it covalently binds FMN to a tag, which is a short, recognizable peptide sequence that is incorporated into the protein of interest (Fig. 6*A*). The tag contains the flavinylation sequence motif and can be as short as seven amino acids. Flavin attachment worked best when the flavin-tag was at the N or C terminus of a target protein, typically leading to full flavin incorporation. The flavinylation sequence motif can also be inserted in a surface loop region of a target protein, yielding a lower degree of flavinylation. The concept of flavin-tagging was demonstrated in *E. coli* and *Saccharomyces cerevisiae* by coexpressing the tagged target protein together with ApbE. Decorating a tagged target protein with FMN can also be performed *in vitro*. This also allowed to label the tagged proteins with flavin derivatives. Thus, proteins with different colors and fluorescent properties were generated (101).

### Flavin-fixing

The majority of flavoproteins contain a dissociable flavin cofactor. Only a minority of enzymes are attached with a covalent flavin cofactor (10). Such covalent flavin-protein linkage has been shown to result in increased protein stability (102, 103, 104), enhanced catalytic activity (104, 105), prevention of cofactor dissociation (106), facilitation of the electron transfer (18), and even modulation of the enzyme's redox properties (105, 107). Inspired by these benefits of covalent flavinylation, we set out to test whether ApbE can be used to turn noncovalent flavoproteins into covalent flavoproteins. For this, we selected several known FMN-containing proteins in which the FMN in not tethered to the protein and for which structures have been elucidated. Analogous to the flavin-tagging approach, we now introduced the necessary mutations in these FMN-containing protein systems in such a way that the recognition sequence motif is introduced with the target threonine positioned close to the phosphate group of the bound FMN (Fig. 6*B*). Upon coexpression of these redesigned proteins with ApbE, it was found that, in most mutant proteins, FMN was covalently incorporated. This novel approach to covalently anchor FMN to an existing flavoprotein was shown to work for four different flavoproteins: a nitroreductase, a mini singlet oxygen generator, an ene reductase, and a light-oxygen-voltage domain protein (108). Pleasingly, the created covalent variants were often found to retain their functionality while the flavin cofactor was irreversibly attached. Clearly, the method is applicable to many different flavoproteins and opens up new avenues for flavoenzyme engineering.

## Conclusions

ApbE is an essential flavin transferase responsible for the covalent attachment of FMN to various proteins. It forms a phosphoester bond between the phosphate group of FMN and a threonine or serine residue. This unique posttranslational modification is widespread in bacteria and plays a critical role in enhancing the stability and catalytic efficiency of flavin-dependent enzyme and electron transfer proteins. This atypical flavin transferase has also been identified in some archaea and eukaryotes, adding to its relevance in biology. Although the functions of many proteins undergoing ApbE-mediated flavinylation remain unknown, future studies are expected to uncover their roles in protein maturation and functionality. Given the distinctive redox properties of flavins, investigating the electron-transfer behavior of flavinylated proteins may provide valuable insights into redox-based biological mechanisms and inspire the development of innovative biotechnologies. Furthermore, the development of potent ApbE inhibitors is a promising avenue for the development of next-generation antibiotics. Such inhibitors could selectively target microorganisms containing ApbE-dependent proteins, such as Na^+^-NQR, RNF complexes, or their analogs, offering a potential strategy to combat the infectious diseases they cause. Finally, ApbE has been shown to be a valuable tool for flavin-tagging and flavin-fixing, enabling direct and selective labeling of proteins and the creation of more efficient and robust flavoproteins, respectively.

## Conflict of interest

The authors declare that they have no conflicts of interest with the contents of this article.
